# Climbing on the La Canna Volcanic Sea Stack to Obtain First-Hand Data on the Tiniest Population of the Critically Endangered Aeolian Wall Lizard *Podarcis raffonei*

**DOI:** 10.3390/ani13142289

**Published:** 2023-07-13

**Authors:** Daniele Salvi

**Affiliations:** Department of Health, Life and Environmental Sciences, University of L’Aquila, 67100 L’Aquila, Italy; danielesalvi.bio@gmail.com

**Keywords:** microinsular endemic, Mediterranean, Lacertidae, genetic diversity, conservation, inbreeding depression

## Abstract

**Simple Summary:**

Information about the status and trends of endangered species populations are vital for conservation actions, yet these data are often limited for those inhabiting remote and difficult-to-access areas. With the technical support of an alpine guide, I climbed on La Canna sea stack, a columnar volcanic pinnacle 70 m high, to gather information on the size, morphology, and genetic variability of the tiniest population of the critically endangered Aeolian wall lizard *Podarcis raffonei*. Results indicate a current population size of about a hundred individuals, a body size of lizards significantly larger than previously known, and a complete lack of genetic diversity. Cephalic malformations observed on all captured individuals indicate the detrimental effect of inbreeding depression and represent a severe threat to the persistence of this population.

**Abstract:**

Among the extant populations of the critically endangered Aeolian wall lizard, the most vulnerable is the one surviving on La Canna, a columnar volcanic stack off the Filicudi Island. Here, I report the results of the first climbing expedition by a biologist on La Canna, that contributed direct observations and updated information on the size, morphology, and genetic variability of this population. Lizard density at the sampling site (a small terrace at 50 m of elevation) was 1.7 m^−2^, twice of a previous estimate. Standard methods for estimating population size are unsuitable for La Canna. An educated guess of about a hundred individuals can be drawn, considering the extent of habitat available on the stack and the number of observed lizards. Lizards on La Canna were not fearless, despite what was reported by alpinists, possibly because of aggressive intraspecific interactions or high environmental temperatures during sampling. Biometric data significantly extend the body size of La Canna’s lizards and indicate that it is not smaller than other *P. raffonei* populations. A complete lack of genetic diversity was found at the mitochondrial *nd4* gene, in line with previous allozyme data and with estimates on other microinsular *Podarcis* populations. The small size of the La Canna population implies severe genetic drift and an extremely high level of inbreeding, as supported by low heterozygosity found across the genome. Detrimental effects of inbreeding depression are evident as cephalic malformations observed in all captured lizards of La Canna and might represent the more immediate threat to the persistence of this population.

## 1. Introduction

The Aeolian wall lizard is perhaps the emblem of a species on the brink of extinction among European herps [1,2]. Surviving on three tiny volcanic stacks and a small peninsula, scattered across the Aeolian Archipelago (North-East Sicily, Italy), this species has an extent of occurrence about half of a hectare, and a total population size probably lower than a couple of thousands of individuals, according to the most recent estimate [3]. Because of the extremely limited area of occupancy, severe population fragmentation, and observed decline, it has been classified as critically endangered by the International Union for the Conservation of Nature [4].

Among the extant populations of this lizard, one of the most vulnerable is the one surviving on La Canna, a columnar volcanic stack rising 71 m above sea level, 1.6 km off Filicudi Island in the N-W sector of the Aeolian archipelago. La Canna represents the remnant lava neck of an eruptive centre that has been almost totally eroded, and that was active from 40 ka until 29 ka [5]. The vertical bare rocks of dark basalts of La Canna provide an exceptionally poor habitat and extreme environmental conditions for lizards, with meagre water and food availability and rock temperatures that exceeds 60 °C during insolation hours in the summer. These vertical cliffs have proven challenging also for herpetologists that never had the opportunity to gather in situ observations on this population. Indeed, since the first discovery of this population in 1973 [6], or in 1972 according to La Greca [7], the only information available for La Canna’s lizards came from professional climbers, who brought down from the stack a few individuals or faecal pellets, to herpetologists for taxonomic assessments and preliminary diet analysis [8,9,10]. In particular, four lizards collected on La Canna in 1979 by the Sicilian alpinist Sergio Cucchiara were taxonomically assessed by Di Palma, and assigned to a new subspecies of the Italian wall lizard, *Podarcis sicula cucchiarai* [8]. And, four lizards collected few years later by another Sicilian alpinist, Giuseppe Maurici, were screened for allozyme loci by Capula in 1990 and assigned to the newly recognised species *Podarcis raffonei* [9,11]. About thirty years later, a few more individuals were observed by the Sicilian alpinists Livia Guarino and Claudia Speciale, who also collected a dozen faecal pellets [10].

Here are reported the results of the first climbing expedition by a biologist on La Canna in 2020, aimed at collecting updated information on the size, morphology, and genetic variability of the tiniest population of the Aeolian wall lizard. The first goal of the expedition was to secure fresh tissue samples from La Canna lizards to generate the first complete genome of *P. raffonei* [12] and population genomic resources for all the extant populations of the species within the framework of the projects ENDEMIXIT and HYBRIND (MUR PRIN projects 201794ZXTL and 2017KLZ3MA, respectively). Biometric and genetic data, as well as observations for populations size estimates, are compared with available data and discussed in relation to future studies.

## 2. Materials and Methods

**Figure 1:**
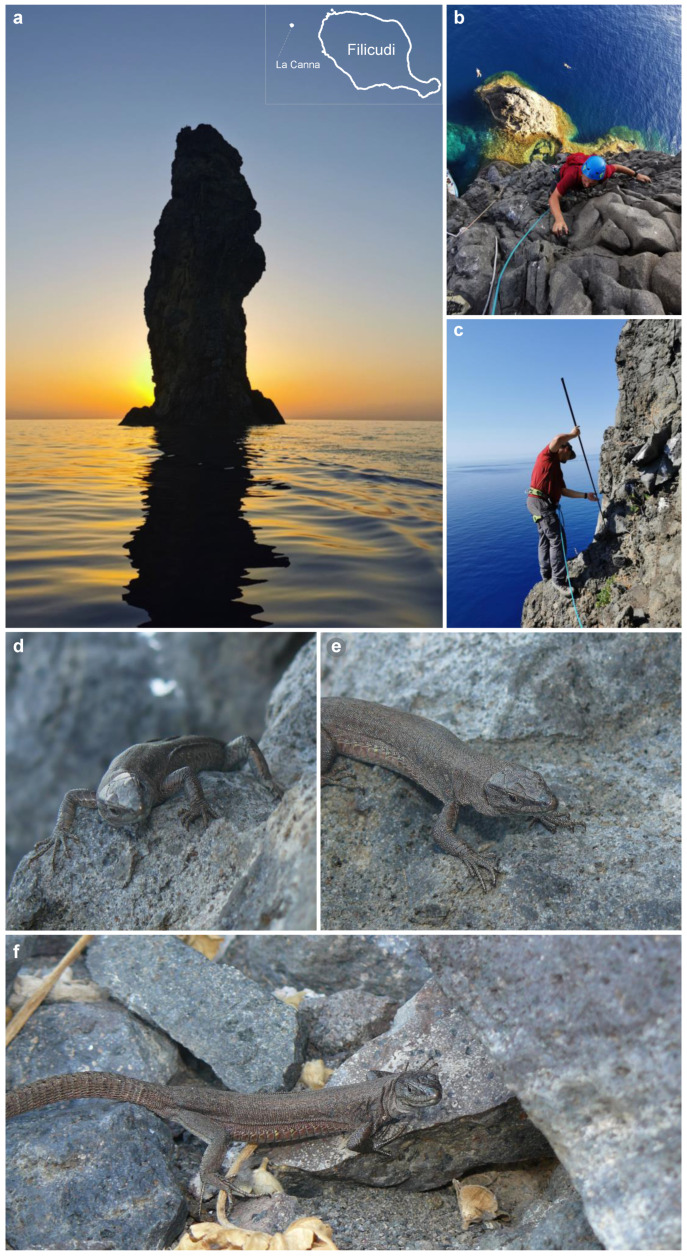
(**a**) The sea stack of La Canna and its position relative to the Filicudi Island (top right inset); (**b**,**c**) climbing and sampling activities on La Canna by the author; (**d**–**f**) *Podarcis raffonei* individuals from La Canna ((**a**–**c**) photos by L. Inzigneri; (**d**–**f**) photos by D. Salvi).

Sample and data collection required climbing the La Canna stack. The expert alpine guide Lorenzo Inzigneri (LI) planned, directed, and coordinated all the climbing activities, from the intensive training sessions of the author (DS) in the Dolomite mountains to the climb planning and execution. The climbing ascent started on the 31st of July 2020 at 6:30 a.m. and was performed in two pitches of about 30 m each, the first toward a small belay edge, and the second up to the sampling spot: a small gently sloping terrace at a 50 m elevation on the eastern side of the stack. LI climbed as lead bolting the route, and DS as top-roped. Sampling was performed by DS constantly secured by LI, who set up a belay station with fixed bolts to sample in safety (Figure 1). Soft fruit pieces were used to attract lizards and maximise detection and capture. Lizards interacted immediately and simultaneously on fruit baits; therefore, using a high number of baits, it was possible to count the number of simultaneously active individuals around each refuge-stone with no pseudo-replication. Lizards were collected by noose from 8.45 to 11.45 then brought down at the base of the stack for data and sample collection. From each lizard, standardised photographs of the whole body (dorsal, ventral and right-lateral sides, cloacal region, and gular region) and of the head, see [13,14], body length measure (snout to vent, SVL), and a small tail tip tissue were collected. A second climbing was performed at 4 pm to bring the lizards back and release them at the capture point.

Total genomic DNA was extracted from alcohol-preserved tail tip tissue following standard high-salt protocols [15]. The mitochondrial NADH dehydrogenase subunit 4 with flanking tRNAs (*nd4*) gene fragment was amplified by polymerase chain reaction (PCR). This fast-evolving marker has been fruitfully used in several phylogeographic and phylogenetic studies on lizards, including *Podarcis* [16,17,18,19,20]. Primers used for PCR and sequencing were ND4 and Leu [21]. Amplifications were conducted in 25 μL volumes, containing 3 μL of 10x reaction buffer (Ecogen), 1.5 µL of MgCl_2_ (50 mM), 0.5 µL dNTPs (10 mM), 0.5 μL each primer (25 mM), 1 U of BIOTAQ DNA polymerase (Bioline), and approximately 50 ng genomic DNA. Amplification conditions consisted of a predenaturing step of 3 min at 94 °C, followed by 35 cycles of a denaturing step of 30 s at 94 °C, annealing at 50 °C for 30 s, extension at 72 °C for 45 s, and the final extension at 72 °C for 5 min. Purification and sequencing of PCR products were carried out by a commercial company, GENEWIZ (Leipzig, Germany).

Sequences were aligned with MAFFT v.7 [22] using the G-INS-I progressive method algorithm, resulting in an alignment of 832 positions. Genetic distance (uncorrected *p*-distance) between samples analysed in this study and sequences obtained from GenBank were calculated with MEGA v.7 [23]. Genetic diversity of the La Canna population was estimated as haplotype diversity (*h*) and nucleotide diversity (*π*) with their standard deviations (SD), using DNASP 5 5.10.01 [24], and compared with values estimated for the same gene fragment in other microinsular *Podarcis* populations compiled from previous phylogeographic studies [20,25,26,27,28,29]. For this purpose, DNA sequence datasets were re-assembled using GenBank accessions and associated haplotype population identifier data within the corresponding publications. Following Goodall-Copestake et al. [30], only populations with ≥5 samples sequenced were included, and sequences were trimmed to the same region (a 611-base homologous region including the 3′ portion of the *nd4* and partial *tRNA^His^*).

Plots shown in this study were generated with ggplot2 [31] in R [32].

## 3. Results and Discussion

**Figure 2:**
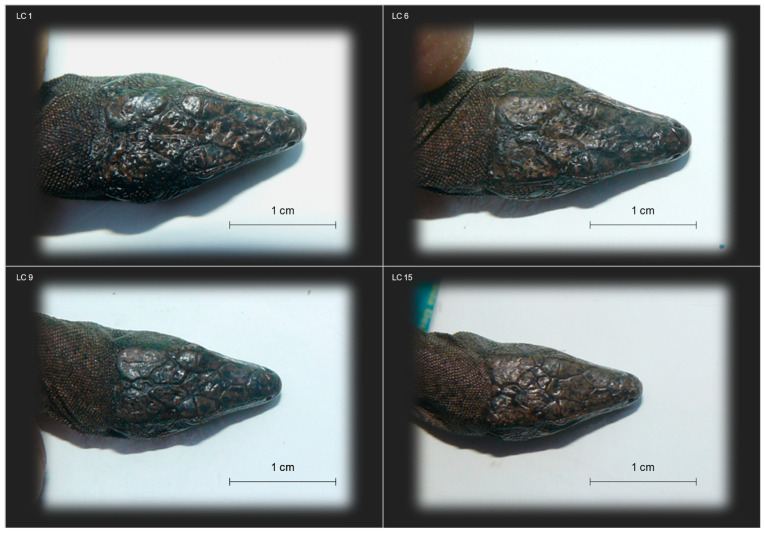
Examples of malformations in cephalic scales (pileus) observed on lizards from La Canna. Malformations vary in types and extent, ranging from deformation of a few scales (e.g., LC 9) to a crumpled-bumpy amorphous structure extending over most of the pileus with loss of the scutellation pattern (e.g., LC 1).

The area that was possible to sample in safety is shaped roughly like a scalene triangle with a base of ~7 m (approximatively estimated using rope lengths as reference), lying on the side of the rock wall, and a high of ~5 m, including the flat and gently sloping portion of the terrace before the cliff overhang, for an approximate surface of 18 m^2^. A few large basaltic stones, and around twenty individuals of the plant *Malva veneta* (ten large plants > 30 cm and 7–8 small plants < 30 cm), offer refuges to lizards. An active nest of the Eleonora’s falcon, *Falco eleonorae*, with a female brooding two eggs, was observed in a rock niche in the southern side of the study area. A total of 31 lizard individuals were observed in a sampled area of ~18 m^2^. Using the same index as [10], individuals found per square meter, the estimated lizard density on the terrace is 1.7 m^−2^, more than double the value estimated by the previous study for the same area (0.8 m^−2^). The use of baits to attract lizards and the higher sampling effort might explain the increased detection efficiency of this study. Sixteen individuals were captured, ten females and six males, all adults; juveniles or subadults were not observed. Fifteen individuals (94%) had mites in one or more of the following three regions: around forelimbs (81%), on the side of the collar (31%), or on the tympanic area (19%). Seven lizards (44%), all females, showed ventral bite marks. Six individuals (38%) showed regenerated tails, and one showed a missing toe (out of thirteen lizards assessed for this feature; 8%). All individuals (100%) showed malformations in cephalic scales (pileus), especially in the parietal area, varying from deformation of a few scales (complete and partial splits) to a crumpled-bumpy amorphous structure extending over most of the pileus with loss of the scutellation pattern (Figure 2).

Based on observations and capture data, I can tentatively discuss previous observations and population size estimates. During the first climbing expedition on La Canna, five alpinists, led by L. Battineschi, observed a couple of fearless lizards and, surprisingly, snakes [6]. Both lizards and snakes immediately aroused the interest of B. Lanza, the lizard from a taxonomic perspective, the presence of snakes for being highly unlikely, as also confirmed by some of the alpinists of the Battineschi’s team [33]. I can exclude that on La Canna, there are conditions for sustaining a snake population, since the suitable available habitat is not sufficient. The fearless behaviour of La Canna lizards should also be reconsidered. While this behaviour is described also by Cucchiara [34] and reported in [10], the lizards I observed were anything but fearless. It was not possible to approach lizards by hands and, before placing fruit baits, the lizard detection rate was extremely low. A possible explanation could be related to the overly hot environmental condition during my observations, which were made under summer insolation maximum (at the very limit of human physiological tolerance). At dangerously high environmental temperatures, avoidance of overheating may force fleeing lizards to select cool destinations and avoid prolonged exposure or activity in a hot microhabitat [35]. Previous observations were made earlier in the season and during the afternoon [10], when the site is fully shaded. However, Cucchiara and Battineschi’s team also climbed on La Canna during the morning hours and, therefore, were fully exposed to solar radiation. An additional factor to consider concerns intraspecific aggressive interactions. On small islets, lizards show higher rates of intraspecific aggression due to increased competition for food, territory, mates, and other resources [36,37,38,39]. Although on La Canna I did not observe proxies of increased aggression (i.e., high rates of bite scars, amputation of toes and tail shedding; [40]), the use of food baits might have enhanced the conditions for aggressive interactions, and could explain the observed wary approach of lizards. Further studies are needed to directly test these hypotheses and disentangle conspecific interactions and tameness towards humans.

The size of the La Canna population was reported to be of 20–60 [1] or 20–30 [41] individuals, based on reasoned guess. The estimate of 82.5 ± 52.5 individuals was proposed in [10] based on three lizards counted during a single visual encounter survey performed by one alpinist along the climbing route. While the tentative nature of this estimate must be acknowledged, the many theoretical and practical limitations of such an approach make this estimate of little use. These includes varying assumption violations, lack of replicated counts, extremely low number of detections, and limited area covered by the linear transect. The assumptions of habitat homogeneity and high lizard detectability across the entire stack are clearly not met. As also noted by Lo Cascio et al. [10], most of the habitat available for the lizards on La Canna is restricted to a small terrace on the eastern side of the stack at a 50 m elevation, where lizard density reaches the maximum. This area was not included in the transect, which in fact only covers a stripe of vertical cliff of bare rock where lizard’s density is the lowest. Detection of lizards in such a vertical cliff while climbing first person is, at best, an elusive target. On a general ground, estimating the size of the La Canna population is challenging because most of the commonly used methods, such as mark-recapture, removal sampling, or N-mixture models, require assumptions that is not possible to meet in this site, and replicates over space and time that are impractical in a columnar sea stack. Currently, we are setting methods for population size estimate based on genomic data and drone imagery [42] but, at present, we can only attempt an educated guess. The observation made in this study of 31 lizards, for a sampling effort of three man-hours in an area of ~18 m^2^, indicates that the latest guess of 20–30 individuals by Capula and Lo Cascio [41] was probably too pessimistic. On the other hand, the extrapolation of the lizard density at the sampling site to the total surface area of the stack would highly overestimate the population size. Considering that a few small habitat patches are available also towards the top of the stack, and lizards are also found on the cliffs [10], the number of lizards on La Canna might be around a hundred individuals.

**Figure 3:**
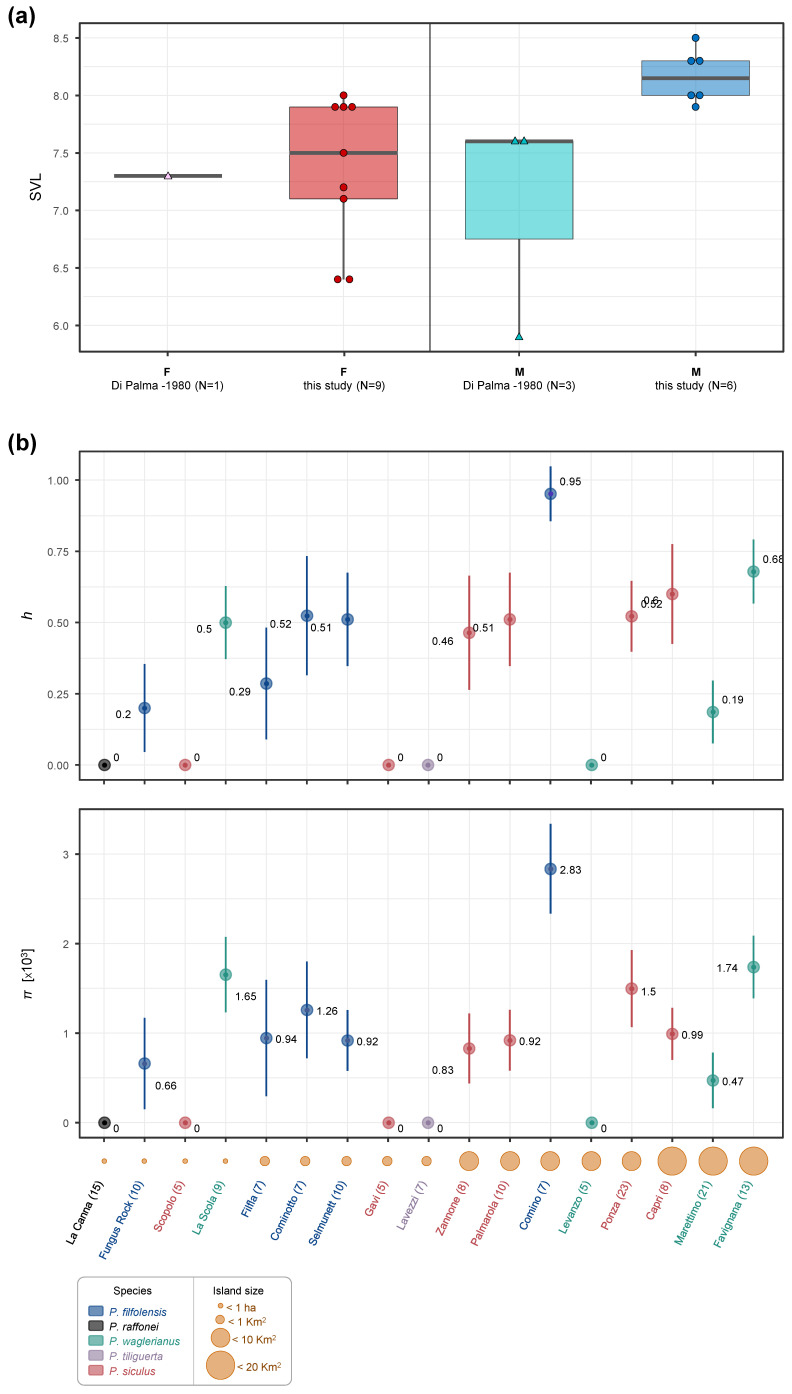
(**a**) Biometric data of the individuals of *Podarcis raffonei* collected in La Canna in this study, compared to measurements available for four individuals by Di Palma [8]. (**b**) Genetic diversity estimates based on the *nd4* mitochondrial gene fragment in the La Canna population, compared with other 16 island populations belonging to the congeneric species *P. waglerianus*, *P. filfolensis*, *P. tiliguerta*, and *P. siculus* compiled from previous studies (see text for details). In the top plot is reported the haplotype diversity (*h* ± SD), and in the bottom plot is reported the nucleotide diversity (*π* ± SD; values are multiplied by 10^3^ to reduce decimal places and increase readability). Along the X-axis, populations are ordered by increasing island size, which is illustrated by circles of different size (smallest circle: islet area 0.2–1 ha; small circle: islet area 2 ha–0.7 km^2^; medium circle: island area 1–7.5 km^2^; large circle: island area 10.3–19.9 km^2^), the number of individuals sequenced per island population (in brackets), and the island name coloured according to the *Podarcis* species present.

Body size measurements (SVL) of females (N = 9) range from 64 to 80 mm (mean = 73.7 mm) and include the value observed for the single female (n.34-M.Z.U.P.: 73 mm) measured by Di Palma [8] (Figure 3a). Body size measures obtained for males (N = 6) range from 79 to 85 mm (mean = 81.7 mm) and are remarkably larger than the values observed in three individuals (n.33-M.Z.U.P.: 76 mm; n.255di tu70-M.Z.U.F.: 76 mm; B.M.-(N.H.): 59 mm; mean = 70.3 mm) by Di Palma [8] (Figure 3a). These results significantly extend the mean and the maximum body size (85 mm) of La Canna’s population and indicate that La Canna’s lizards are not smaller than those of Strombolicchio (SVL = 73.4–83.7 mm) as previously reported [43]. At this regard the value of 73.1 mm used as upper limit in the identification key of the subspecies of La Canna by Capula and Lo Cascio [43] is not confirmed either by this study or Di Palma [8] and not useful to discriminate current populations of *P. raffonei* (see also [3]). 

A single haplotype was found at the gene fragment in the sequenced individuals. The haplotype found in the La Canna population is five substitutions different from the haplotype sequenced by Salvi et al. [25,44] in two individuals of *P. raffonei* from the population of Strombolicchio, which corresponds to a low intraspecific divergence (0.6% uncorrected genetic distance) compared to other wall lizards [20,25,26,28,45]. The occurrence of a single haplotype in La Canna translates to a haplotype and nucleotide diversity equal to zero. Such a lack of genetic variability was also found at allozyme loci screened in four individuals from La Canna by Capula [46]. Comparisons of *nd4* genetic diversity estimates was possible for 16 insular populations of five congeneric species (Figure 3b), including the closely related *P. waglerianus* and *P. filfolensis*, as well as for the species *P. tiliguerta* and *P. siculus* that belong to distinct *Podarcis* clades [44,47]. The number of population-level samples per species ranged from 1 to 6, and the number of individuals per sample ranged from the minimum cutoff of 5 to 23. Haplotype diversity ranged from 0 to 0.95 (*h* mean = 0.37; *h* median = 0.48), and nucleotide diversity ranged from 0 to 0.00283 (*π* mean = 0.00093; *π* median = 0.00092). Lack of haplotype and nucleotide diversity was also observed in four populations of three *Podarcis* species inhabiting a sea stack (<1 ha, Scopolo), two tiny islets (<1 km^2^, Lavezzi and Gavi). and a relatively larger island (~6 km^2^, Levanzo), although in all these cases, the population size was smaller (N < 7) than La Canna (N = 15). On the other hand, five populations inhabiting the tiny (<1 km^2^) or tiniest (<1 ha, La Scola and Fungus Rock) islets show from low to high haplotype and nucleotide diversity (i.e., values below or above the median, respectively). Therefore, besides the limitation of estimates based on a short gene fragment, and of uneven sampling strategies (i.e., unknown numbers of sites sampled per island), in addition to island size, other factors may contribute to the lack of genetic diversity of the La Canna and other *Podarcis* populations. Probably, the extremely poor and reduced habitat available on La Canna (i.e., lowest carrying capacity) is the main determinant factor for the small size of this population, resulting in severe genetic drift and inbreeding that, in turn, can result in reduced fertility, hatchling success, offspring survival, and individual fitness [48]. This is further supported by the very low heterozygosity found at the genome level in one individual of La Canna sampled in this study [12]. The highest prevalence of cephalic malformations observed in lizards of La Canna is most likely an expression of the detrimental effect of inbreeding depression, as observed in other reptiles [48,49,50,51]. In some specimens, the deformation is restricted to the parietal area, and it seems symmetric, whereas in others it is asymmetric, and involves few to many cephalic scales. Both symmetric and asymmetric malformations indicate perturbations of developmental pathways due to increased homozygosity for deleterious alleles, as well as to stressful conditions during egg incubation [52,53,54,55,56].

Beside the collapse of the La Canna stack itself, deleterious effects of low genetic diversity and inbreeding depression might represent the more immediate threat to the persistence of La Canna population.

## 4. Conclusions

The tiny population size of La Canna is associated with a collapse of genetic variability and inbreeding depression. Drone-based remote sensing imagery may provide a suitable and cost-effective approach for monitoring the size and the status of this population. Research in progress on the effect of deleterious mutations associated with inbreeding depression (MUR PRIN 201794ZXTL to Giorgio Bertorelle, University of Ferrara) will provide key information for guiding conservation actions that include ex situ strategies and genetic rescue through outbreeding programs.

## Data Availability

DNA sequence data generated in this study are available in GenBank.

## References

[1] Capula M., Luiselli L., Bologna M.A., Ceccarelli A. (2002). The decline of the Aeolian wall lizard, *Podarcis raffonei*: Causes and conservation proposals. Oryx.

[2] Gippoliti S., Capula M., Ficetola G.F., Salvi D., Andreone F. (2017). Threatened by Legislative Conservationism? The Case of the Critically Endangered Aeolian Lizard. Front. Ecol. Evol..

[3] Ficetola G.F., Silva-Rocha I., Carretero M.A., Vignoli L., Sacchi R., Melotto A., Scali S., Salvi D. (2021). Status of the largest extant population of the critically endangered Aeolian lizard *Podarcis raffonei* (Capo Grosso, Vulcano island). PLoS ONE.

[4] IUCN (2009). Podarcis raffonei. The IUCN Red List of Threatened Species.

[5] Lucchi F., Santo A.P., Tranne C.A., Peccerillo A., Keller J., Lucchi F., Peccerillo A., Keller J., Tranne C.A., Rossi P.L. (2013). Volcanism, magmatism, volcano-tectonics and sea-level fluctuations in the geological history of Filicudi (western Aeolian archipelago). The Aeolian Islands Volcanoes.

[6] Bettineschi L., Jacchini F., Jacchini C., Pala M., Pironi L. (1973). Cinque guide di Macugnaga sopra “La Canna” di Filicudi nelle Isole Eolie. Riv. Mens. CAI.

[7] La Greca G. (2021). Scalare col mare attorno: La Canna di Filicudi. In Alto—Cronaca della Società Alpina Friulana.

[8] Di Palma M.G. (1980). La lucertola del faraglione “La Canna” nelle Isole Eolie: *Podarcis sicula cucchiarai* subsp. nova (Reptilia, Lacertidae). Nat. Sicil..

[9] Capula M. (1994). Genetic variation and differentiation in the lizard, *Podarcis wagleriana* (Reptilia: Lacertidae). Biol. J. Linn. Soc..

[10] Lo Cascio P., Grita F., Guarino L., Speciale C. (2014). A little is better than none: New insights into the natural history of the Aeolian wall lizard *Podarcis raffonei* from La Canna stack (Squamata Sauria). Nat. Sicil..

[11] Capula M. (1990). Ricerche sulla Struttura Genetica di *Podarcis sicula*, *P. wagleriana* e *P. filfolensis* (Reptilia: Lacertidae): Aspetti Tassonomici ed Evolutivi. Ph.D. Thesis.

[12] Gabrielli M., Benazzo A., Biello R., Ancona L., Fuselli S., Iannucci A., Balacco J., Mountcastle J., Tracey A., Ficetola G.F. (2023). A high-quality reference genome for the critically endangered Aeolian wall lizard, *Podarcis raffonei*. J. Hered..

[13] Kaliontzopoulou A., Carretero M.A., Llorente G.A. (2008). Head shape allometry and proximate causes of head sexual dimorphism in *Podarcis* lizards: Joining linear and geometric morphometrics. Biol. J. Linn. Soc..

[14] Piras P., Salvi D., Ferrara G., Maiorino L., Delfno M., Pedde L., Kotsakis T. (2011). The role of post-natal ontogeny in the evolution of phenotypic diversity in *Podarcis* lizards. J. Evol. Biol..

[15] Sambrook J., Fritsch E.F., Maniatis T. (1989). Molecular Cloning: A Laboratory Manual.

[16] Salvi D., Harris D.J., Bombi P., Carretero M.A., Bologna M.A. (2010). Mitochondrial phylogeography of the Bedriaga’s rock lizard, *Archaeolacerta bedriagae* (Reptilia: Lacertidae) endemic to Corsica and Sardinia. Mol. Phylogenet. Evol..

[17] Salvi D., Harris D.J., Perera A., Bologna M.A., Carretero M.A. (2011). Preliminary survey on genetic variation within the pygmy algyroides, *Algyroides fitzingeri*, across Corsica and Sardinia. Amphibia-Reptilia.

[18] Mendes J., Harris D.J., Carranza S., Salvi D. (2016). Evaluating the phylogenetic signal limit from mitogenomes, slow evolving nuclear genes, and the concatenated approach. New insights into the Lacertini radiation using fast evolving nuclear genes and species trees. Mol. Phylogenet. Evol..

[19] Mendes J., Harris D.J., Carranza S., Salvi D. (2017). Biogeographical crossroad at the Pillars of Hercules: Evolutionary history of *Psammodromus* lizards in space and time. J. Biogeogr..

[20] Senczuk G., Harris D.J., Castiglia R., Litsi Mizan V., Colangelo P., Canestrelli D., Salvi D. (2019). Evolutionary and demographic correlates of Pleistocene coastline changes in the Sicilian wall lizard *Podarcis wagleriana*. J. Biogeogr..

[21] Arévalo E., Davis S.K., Sites J.W.J. (1994). Mitochondrial DNA sequence divergence and phylogenetic relationships among eight chromosome races of the *Sceloporus grammicus* complex (Phrynosomatidae) in Central Mexico. Syst. Biol..

[22] Katoh K., Standley D.M. (2013). MAFFT multiple sequence alignment software version 7: Improvements in performance and usability. Mol. Biol. Evol..

[23] Kumar S., Stecher G., Tamura K. (2016). MEGA7: Molecular Evolutionary Genetics Analysis version 7.0 for bigger datasets. Mol. Biol. Evol..

[24] Librado P., Rozas J. (2009). DnaSP v5. a software for comprehensive analysis of DNA polymorphism data. Bioinformatics.

[25] Salvi D., Schembri P.J., Sciberras A., Harris D.J. (2014). Evolutionary history of the Maltese wall lizard *Podarcis filfolensis*: Insights on the ‘Expansion-Contraction’ model of the Pleistocene biogeography. Mol. Ecol..

[26] Salvi D., Pinho C., Harris D.J. (2017). Digging up the roots of an insular hotspot of genetic diversity: Decoupled mito-nuclear histories in the evolution of the Corsican Sardinian endemic lizard *Podarcis tiliguerta*. BMC Evol. Biol..

[27] Senczuk G., Havenstein K., Milana V., Ripa C., De Simone E., Tiedemann R., Castiglia R. (2018). Spotlight on islands: On the origin and diversification of an ancient lineage of the Italian wall lizard *Podarcis siculus* in the western Pontine Islands. Sci. Rep..

[28] Senczuk G., Castiglia R., Colangelo P., Delaugerre M., Corti C. (2019). The role of island physiography in maintaining genetic diversity in the endemic Tyrrhenian wall lizard (*Podarcis tiliguerta*). J. Zool..

[29] Buglione M., Petrelli S., Maselli V., Trapanese M., Salvemini M., Aceto S., Di Cosmo A., Fulgione D. (2019). Fixation of genetic variation and optimization of gene expression: The speed of evolution in isolated lizard populations undergoing Reverse Island Syndrome. PLoS ONE.

[30] Goodall-Copestake W.P., Tarling G.A., Murphy E.J. (2012). On the comparison of population-level estimates of haplotype and nucleotide diversity: A case study using the gene *cox1* in animals. Heredity.

[31] Wickham H. (2011). ggplot2. Wiley Interdisciplinary Reviews: Computational Statistics 3.2.

[32] R Core Team (2022). R: A Language and Environment for Statistical Computing.

[33] Lanza B. (1973). Gli Anfibi e i Rettili delle isole circumsiciliane. Lav. Soc. Ital. Biogeogr..

[34] Cucchiara S. (1975). La seconda scalata alla “Canna” di Filicudi. Scarpone.

[35] Cooper W.E. (2000). Effect of temperature on escape behaviour by an ectothermic vertebrate, the keeled earless lizard (*Holbrookia propinqua*). Behaviour.

[36] Pafilis P., Meiri S., Foufopoulos J., Valakos E. (2009). Intraspecific competition and high food availability are associated with insular gigantism in a lizard. Naturwissenschaften.

[37] Vervust B., Van Dongen S., Grbac I., Van Damme R. (2009). The mystery of the missing toes: Extreme levels of natural mutilation in island lizard populations. Funct. Ecol..

[38] Raia P., Guarino F.M., Turano M., Polese G., Rippa D., Carotenuto F., Monti D.M., Cardi M., Fulgione D. (2010). The blue lizard spandrel and the island syndrome. BMC Evol. Biol..

[39] Brock K.M., Bednekoff P.A., Pafilis P., Foufopoulos J. (2014). Evolution of antipredator behavior in an island lizard species, *Podarcis erhardii* (Reptilia: Lacertidae): The sum of all fears?. Evolution.

[40] Donihue C.M., Brock K.M., Foufopoulos J., Herrel A. (2016). Feed or fight: Testing the impact of food availability and intraspecific aggression on the functional ecology of an island lizard. Funct. Ecol..

[41] Capula M., Lo Cascio P., Sindaco R., Doria G., Razzetti E., Bernini F. (2006). Podarcis *raffonei* (Mertens, 1953). Atlas of Italian Amphibians and Reptile.

[42] Dubos N., Porcel X., Roesch M.A., Claudin J., Pinel R., Probs J.-M., Deso G. (2023). A bird’s-eye view: Evaluating drone imagery for the detection and monitoring of endangered and invasive day gecko species. Biotropica.

[43] Capula M., Lo Cascio P., Corti C., Capula M., Luiselli L., Razzetti E., Sindaco R. (2011). Podarcis *raffonei* (Mertens, 1952). Fauna d’Italia, Reptilia.

[44] Salvi D., Pinho C., Mendes J., Harris D.J. (2021). Fossil-calibrated time tree of *Podarcis* wall lizards provides limited support for biogeographic calibration models. Mol. Phylogenet. Evol..

[45] Salvi D., Harris D.J., Kaliontzopoulou A., Carretero M.A., Pinho C. (2013). Persistence across Pleistocene ice ages in Mediterranean and extra-Mediterranean refugia: Phylogeographic insights from the common wall lizard. BMC Evol. Biol..

[46] Capula M. (2004). Low genetic variation in a critically endangered Mediterranean lizard: Conservation concerns for *Podarcis raffonei* (Reptilia, Lacertidae). Ital. J. Zool..

[47] Yang W., Feiner N., Pinho C., Kaliontzopoulou A., While G.M., Harris D.J., Salvi D., Uller T. (2021). Extensive introgression and mosaic genomes of endemic Mediterranean lizards. Nat. Commun..

[48] Lindsay W.R., Madsen T., Wapstra E., Lillie M., Loeb L., Ujvari B., Olsson M. (2020). Long term effects of outbreeding: Experimental founding of island population eliminates malformations and improves hatching success in sand lizards. Biol. Conserv..

[49] Sarre S., Dearn J.M. (1991). Morphological variation and fluctuating asymmetry among insular populations of the sleepy lizard, *Trachydosaurus-Rugosus* Gray (Squamata, Scincidae). Aust. J. Zool..

[50] Olsson M., Gullberg A., Tegelström H. (1996). Malformed offspring, sibling matings, and selection against inbreeding in the sand lizard (*Lacerta agilis*). Evol. Biol..

[51] Abramjan A., Frýdlová P., Jančúchová-Lásková J., Suchomelová. P., Landová E., Yavruyan E., Frynta D. (2019). Comparing developmental stability in unisexual and bisexual rock lizards of the genus *Darevskia*. Evol. Dev..

[52] Palmer A.R., Strobeck C. (1986). Fluctuating asymmetry: Measurements, analysis, patterns. Annu. Rev. Ecol. Syst..

[53] Leary R.F., Allendorf F.W. (1989). Fluctuating asymmetry as an indicator of stress: Implications for conservation biology. Trends Ecol. Evol..

[54] Paredes U., Radersma R., Cannell N., While G.M., Uller T. (2016). Low incubation temperature induces DNA hypomethylation in lizard brains. J. Exp. Zool..

[55] Brown G.P., Madsen T., Dubey S., Shine R. (2017). The causes and ecological correlates of head scale asymmetry and fragmentation in a tropical snake. Sci. Rep..

[56] Idrisova L.A. (2018). The effect of incubation temperature on deviations of pholidosis and malformations in grass snake *Natrix natrix* (L. 1758) and sand lizard *Lacerta agilis* (L. 1758). KnE Life Sci..

